# Changes in Healthcare Utilization During the COVID-19 Pandemic and Potential Causes—A Cohort Study From Switzerland

**DOI:** 10.3389/ijph.2023.1606010

**Published:** 2023-07-26

**Authors:** Erika Harju, Alexandre Speierer, Katharina Tabea Jungo, Sara Levati, Stéphanie Baggio, Stefano Tancredi, Nazihah Noor, Pierre-Yves Rodondi, Stéphane Cullati, Medea Imboden, Dirk Keidel, Melissa Witzig, Irène Frank, Philipp Kohler, Christian Kahlert, Luca Crivelli, Rebecca Amati, Emiliano Albanese, Marco Kaufmann, Anja Frei, Viktor von Wyl, Milo A. Puhan, Nicole Probst-Hensch, Gisela Michel, Nicolas Rodondi, Patricia Chocano-Bedoya

**Affiliations:** ^1^ Faculty of Health Sciences and Medicine, University of Lucerne, Lucerne, Switzerland; ^2^ School of Health Sciences, ZHAW Zurich University of Applied Sciences, Winterthur, Switzerland; ^3^ Clinical Trial Unit, Cantonal Hospital Lucerne, Lucerne, Switzerland; ^4^ Institute of Primary Health Care (BIHAM), University of Bern, Bern, Switzerland; ^5^ Department of Business Economics, Health and Social Care, University of Applied Sciences and Arts of Southern Switzerland, Manno, Switzerland; ^6^ Population Health Laboratory (#PopHealthLab), Department of Community Health, Faculty of Science and Medicine, University of Fribourg, Fribourg, Switzerland; ^7^ Institute of Family Medicine, University of Fribourg, Fribourg, Switzerland; ^8^ Quality of Care Service, Geneva University Hospitals, Geneva, Switzerland; ^9^ Department of Epidemiology and Public Health, Swiss Tropical and Public Health Institute (Swiss TPH), Allschwil, Switzerland; ^10^ Department of Public Health, University of Basel, Basel, Switzerland; ^11^ Department of Infectious Diseases and Hospital Epidemiology, Cantonal Hospital St. Gallen, St. Gallen, Switzerland; ^12^ Infectious Diseases and Hospital Epidemiology, Children’s Hospital of Eastern Switzerland, St. Gallen, Switzerland; ^13^ Institute of Public Health, Faculty of Biomedical Sciences, Università della Svizzera Italiana, Lugano, Switzerland; ^14^ Epidemiology, Biostatistics and Prevention Institute, Faculty of Medicine, University of Zurich, Zurich, Switzerland; ^15^ Institute for Implementation Science in Health Care, Faculty of Medicine, University of Zurich, Zürich, Switzerland; ^16^ Department of General Internal Medicine, Inselspital, Bern University Hospital, University of Bern, Bern, Switzerland

**Keywords:** COVID-19, digital follow-up, healthcare utilization, healthcare delivery, population-based study

## Abstract

**Objectives:** To describe the frequency of and reasons for changes in healthcare utilization in those requiring ongoing treatment, and to assess characteristics associated with change, during the second wave of the pandemic.

**Methods:** Corona Immunitas e-cohort study (age ≥20 years) participants completed monthly questionnaires. We compared participants reporting a change in healthcare utilization with those who did not using descriptive and bivariate statistics. We explored characteristics associated with the number of changes using negative binomial regression.

**Results:** The study included 3,190 participants from nine research sites. One-fifth reported requiring regular treatment. Among these, 14% reported a change in healthcare utilization, defined as events in which participants reported that they changed their ongoing treatment, irrespective of the reason. Reasons for change were medication changes and side-effects, specifically for hypertension, or pulmonary embolism treatment. Females were more likely to report changes [Incidence Rate Ratio (IRR) = 2.15, *p* = 0.002]. Those with hypertension were least likely to report changes [IRR = 0.35, *p* = 0.019].

**Conclusion:** Few of those requiring regular treatment reported changes in healthcare utilization. Continuity of care for females and chronic diseases besides hypertension must be emphasized.

## Introduction

Due to the COVID-19 pandemic, healthcare providers and policymakers had to reorganize their care management to allocate sufficient resources for the treatment of persons affected by COVID-19, while simultaneously trying to limit contamination risks and continuing to care for non-COVID-19 patients. These efforts have resulted in a decrease in healthcare service provision and healthcare utilization for non-COVID-19-related routine medical care at the beginning of the pandemic [1, 2].

Few studies have examined the specific reasons behind missed appointments and lack of care [1, 3]. It has been hypothesized that lockdown policies, stay-at-home mandates, and social distancing may have contributed to people avoiding or delaying consultation and treatment [1, 3, 4]. Fear of infection when seeking hospital care might be another explanation for reduced access to healthcare services [5]. The worry of adding to the burden on healthcare staff, and hospital resources, rendering a sense of solidarity, could also lead to missed appointments and lack of care [6, 7].

In Switzerland, although a complete lockdown did not occur, the Federal Office of Public Health (FOPH) issued recommendations to stay at home [8]. Reduced provision of health services has also significantly impacted the frequency and type of healthcare utilization among individuals requiring regular follow-up care and monitoring [4]. Some studies have reported an increase in health service utilization related to telemedicine [9, 10]. In a previous study, researchers used a database of electronic medical records to model changes in weekly consultations from patients with diabetes, hypertension, and cardiovascular diseases (CVD), comparing the first wave of the pandemic with the previous year. The authors found the number of weekly visits to be 17.2% lower than expected, based on the numbers from 2019 [4]. Additionally, the reduction in the measurements of relevant health parameters was more pronounced than in consultation counts. Vulnerable populations, such as patients with low levels of education, chronically ill, or women have been found to forgo healthcare [11].

Previous studies on healthcare utilization during the pandemic in Switzerland were limited to specific types of care such as general practices (GP) and psychological counseling, focused on patients in need of consistent disease-specific monitoring, such as diabetes, CVD, hypertension, cancer treatment, chronic obstructive pulmonary disease (COPD), and post-transplant, or included specific vulnerable populations [2, 4, 6, 10–13]. Therefore, they are not representative of healthcare utilization by the general population in Switzerland. In addition, patient perspectives of healthy and low-risk individuals, on why healthcare utilization during the pandemic has changed, are scarce. Furthermore, most previous studies were limited to the first months of the pandemic from March 2020 to August 2020 [1].

The main objective of this study was to assess the impact of the pandemic on the utilization of healthcare services in the general Swiss population between September 2020 and February 2021 (the second wave of the pandemic). We first aimed to describe healthcare utilization during the COVID-19 pandemic in the general population, specifically the frequency of changes and reasons therefore in those needing regular treatment, and healthcare utilization in those not needing regular treatment. Second, we aimed to assess the characteristics of those who reported changes in regular healthcare utilization, and characteristics associated with the frequency of changes in regular healthcare utilization.

## Methods

### National Research Project

Corona Immunitas is a Swiss-wide research program of coordinated population-based, seroprevalence studies coordinated by the Swiss School of Public Health (SSPH+) and implemented in Swiss cantons of all language regions since spring 2020. Until 2022, 13 sites participated in the program: Basel-City (BS), Basel-Land (BL), Bern (BE), Fribourg (FR), Eastern Switzerland [St. Gallen (SG) and Grisons (GR)], Geneva (GE), Lucerne (LU), Neuchatel (NE), Ticino (TI), Valais (VS), Vaud (VD) and Zurich (ZH). The program’s main objective was to measure the spread of COVID-19 infections in the general population by measuring antibodies in individuals randomly selected by the Swiss Federal Statistical Office (SFSO) across Switzerland [14]. All participating sites used standardized methods and questionnaires to ensure comparability. The Corona Immunitas research program has been described in more detail elsewhere [14].

The study was conducted according to the Declaration of Helsinki guidelines. The ethics committees of the various cantons approved this study: BS and BL, BASEC No 2020-00927; BE, GR, SG, FR, LU, NE, VS and ZH, BASEC No. 2020-01247; GE, BASEC No. 2020-00881; TI, BASEC No. 2020-01514; VD, BASEC No. 2020-00887.

### Study Population and Procedure

Eligible individuals from age-stratified random samples of the general Swiss population (20–64 years and 65+ years) provided by the SFSO were recruited. Individuals received an invitation letter, including study information, informed consent forms and a pre-paid return envelope. In the first part, participants completed a baseline questionnaire, that included sociodemographic and health-related information, such as the presence of chronic conditions (cancer, diabetes, immunocompromised, hypertension, CVD, chronic respiratory disease) [15]. Participants attended a baseline visit for blood collection to measure seroprevalence. In the second part, which required separate informed consent, they participated in the electronic Corona Immunitas Follow-Up (eCohort) for a duration of 6–12 months. This longitudinal part of the study consisted of weekly and monthly questionnaires to assess self-reported population-based information related to COVID-19 such as risk behavior, adherence to preventive measures, changes in employment, and many more [16]. The data was collected using REDCap (Research Electronic Data Capture), a secure, web-based software platform [17, 18].

For this study, we used available longitudinal data from nine research sites BS, BL, BE, FR, GR, LU, NE, SG, and ZH collected between September 2020 and March 2021, which corresponded to a period of high incidence of COVID-19 infections [19]. In total, 6,252 participants agreed to be part of the eCohort. Participants were ≥20 years old, provided written informed consent, and completed the baseline questionnaire and at least one of the monthly questionnaires.

### Measurements

**FIGURE 1:**
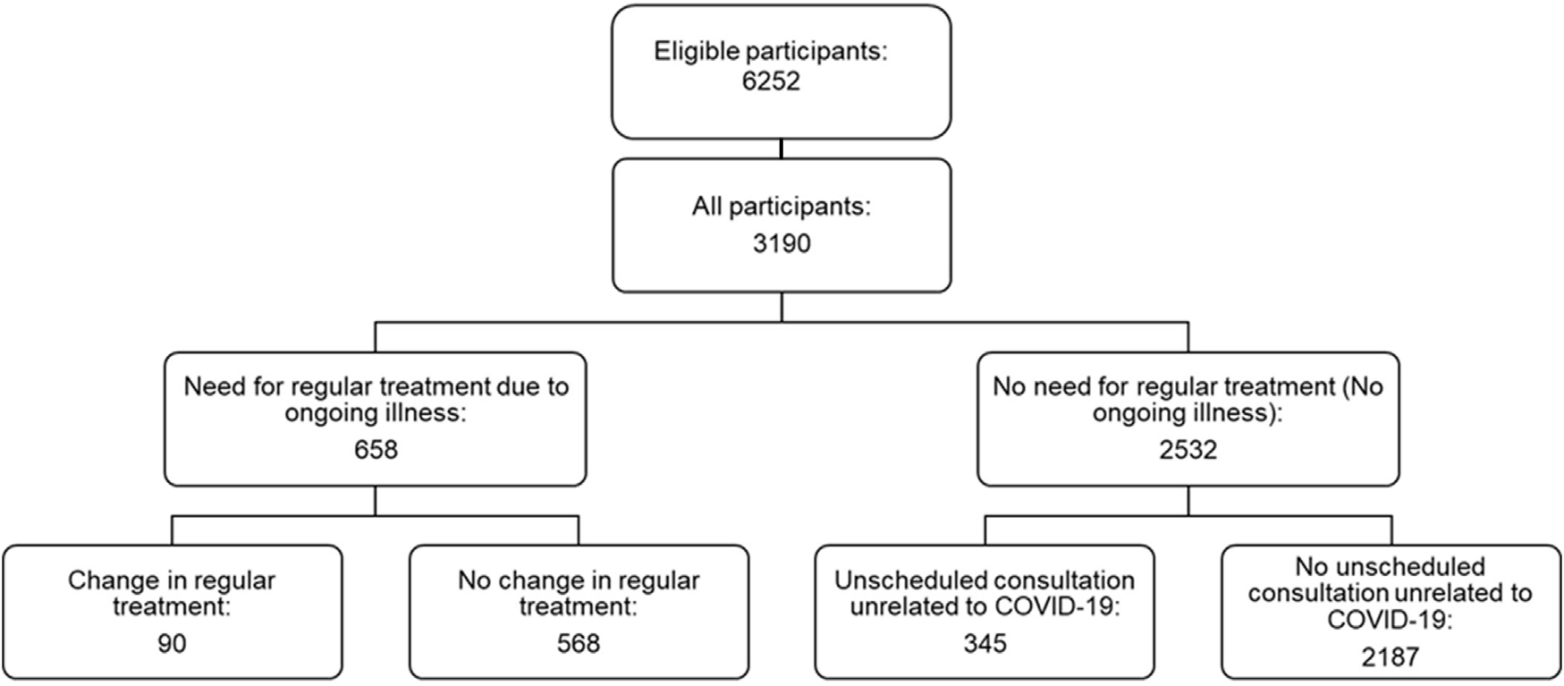
Flow chart of study population, Corona Immunitas eCohort of nine centers (Switzerland, September 2020–February 2021).

Monthly, participants self-reported if they needed regular treatment for an ongoing illness (“Yes,” “No”; Figure 1 and Table 1, and Supplemental Material Monthly Questionnaire). If so, participants were asked if they had changed their regular treatment during the previous month “Have you changed your ongoing medical treatment during the past month?”. Several answers were possible (“No, I have not changed my treatment,” “Yes, I have had problems obtaining my usual treatment,” “Yes, I have stopped my treatment to not risk aggravating a potential Coronavirus infection,” “Yes, for another reason”). We assessed the reasons for changes in regular treatment with the open answer possibility “Yes, for another reason.” We defined “change in healthcare utilization” as events in which participants reported that they changed their regular treatment, irrespective of the reason. The number of changes in those needing regular treatment was counted for each participant for the duration of the study.

**TABLE 1:** Characteristics of study population, Corona Immunitas eCohort of nine centers (Switzerland, September 2020–February 2021).

All participants									
	No regular treatment				Regular treatment				
					No change in treatment		Change in treatment		
	N = 3,190		N = 2,532		N = 568		N = 90		
	Mean	SD	Mean	SD	Mean	SD	Mean	SD	*p*-value
Age at study (years)	54.22	16.09	53.01	16.27	58.83	14.58	59.14	13.73	0.850^b^
BMI	25.07	5.09	24.94	5.18	25.46	4.65	25.98	5.16	0.337^b^
	**n**	**%**	**n**	**%**	**n**	**%**	**n**	**%**	***p*-value**
Age group (years)									0.522^c^
20–64	2,113	66.2	1,746	69.0	314	55.3	53	58.9	
65+	1,077	33.8	786	31.0	254	44.7	37	41.1	
Gender									0.002^d^
Female	1,620	50.8	1,260	49.8	297	52.3	63	70.0	
Male	1,568	49.2	1,270	50.2	271	47.7	27	30.0	
Other	2	0.1	2	0	0	0	0	0	
Language region									0.860^c^
French-speaking	603	18.9	447	17.7	134	23.6	22	24.4	
German-speaking	2,587	81.1	2,085	82.3	434	76.4	68	75.6	
Citizenship^a^									0.030^c^
Swiss	2,548	79.9	2,009	79.3	474	83.5	65	72.2	
Swiss and other	269	8.4	213	8.4	44	7.7	12	13.3	
Other	366	11.5	305	12.0	48	8.5	13	14.4	
Highest education achieved^a^									0.690^d^
Primary	117	3.7	89	3.5	25	4.4	3	3.3	
Secondary	1,472	46.1	1,194	47.2	243	42.8	35	38.9	
Tertiary	1,587	49.7	1,238	48.9	297	52.3	52	57.8	
Employment status^a^									0.710^d^
Unemployed	95	3.0	63	2.5	26	4.6	6	6.7	
Employed (part- or full-time)	1,849	58.0	1,546	61.1	260	45.8	43	47.8	
Retired only	1,001	31.4	729	28.8	238	41.9	34	37.8	
Studying only	81	2.5	68	2.7	10	1.8	3	3.3	
Other	157	4.9	121	4.8	32	5.6	4	4.4	
Current monthly (gross) household income^a^									0.912^d^
≤6,000 CHF	996	31.2	770	30.4	194	34.2	32	35.6	
6,001–≤12,000 CHF	1,457	45.7	1,163	45.9	258	45.4	36	40.0	
12,001–≤18,000 CHF	402	12.6	323	12.8	68	12.0	11	12.2	
>18,001 CHF	161	5.0	128	5.1	29	5.1	4	4.4	
Chronic health condition									0.044^d^
No chronic condition	2,242	70.3	1917	75.7	279	49.1	46	51.1	
Only cancer	35	1.1	21	0.8	12	2.1	2	2.2	
Only diabetes	40	1.3	21	0.8	16	2.8	3	3.3	
Only immunocompromised	47	1.5	21	0.8	24	4.2	2	2.2	
Only hypertension	371	11.6	280	11.1	86	15.1	5	5.6	
Only cardiovascular disease	64	2.0	46	1.8	16	2.8	2	2.2	
Only chronic respiratory disease	119	3.7	86	3.4	23	4.0	10	11.1	
More than one chronic condition	272	8.5	140	5.5	112	19.7	20	22.2	
Household size (additional persons)^a^									0.721^d^
None	502	15.7	370	14.6	113	19.9	19	21.1	
1 Person	1,467	46.0	1,135	44.8	284	50.0	48	53.3	
2 Persons	510	16.0	417	16.5	83	14.6	10	11.1	
3 Persons	486	15.2	420	16.6	57	10.0	9	10.0	
4+ Persons	207	6.5	175	7.5	29	5.1	3	3.3	
Smoking status (cigarettes)^a^									0.888^c^
Current smoker	502	15.8	400	15.8	88	15.5	14	15.5	
Former smoker	843	26.4	630	24.9	184	32.4	29	32.2	
Never smoked	1,843	57.8	1,501	59.3	295	51.9	47	52.2	
Previous SARS-CoV-2 PCR test(s) (baseline)^a^									0.184^c^
No PCR test	2,304	72.2	1,846	72.9	403	71.0	55	61.1	
Yes, tested positive	160	5	131	5.2	23	4.0	6	6.7	
Yes, tested negative	714	22.4	550	21.7	137	24.1	27	30	

Abbreviations: Swiss Francs, CHF; Number, N; Severe acute respiratory syndrome coronavirus, 2SARS-CoV-2; Polymerase chain reaction, PCR.

^a^ Contains missing values.

^b^ *p*-value from Two-sample *t*-test within those who reported needing regular treatment.

^c^ *p*-value from Pearson’s chi-squared test within those who reported needing regular treatment.

^d^ *p*-value from Fisher’s exact test within those who reported needing regular treatment.

We further assessed fear about potential COVID-19 infection during regular healthcare service utilization using a 5-point-Likert scale (1 “Yes, very afraid” to 5 “No, not afraid”) as a reason for change in healthcare utilization.

Participants who reported not needing regular treatment (e.g., not having an ongoing illness) were asked about unscheduled healthcare consultations unrelated to COVID-19 instead: “During the past month, did you need to see a health professional for an acute health problem not related to the Coronavirus and not part of an ongoing treatment?” (“Yes”, “No”). Possible answers for healthcare services utilization regarding that consultation were: “Using telemedicine,” “At home,” “In a doctor’s office (GP),” “In a hospital department,” “In hospital emergency rooms,” and “Other.” In this group, we also assessed fear of contracting COVID-19 during that consultation using a 5-point-Likert scale (1 “Yes, very afraid” to 5 “No, not afraid”). We dichotomized the fear variable by combining values 1 and 2 into 1 “Yes, afraid” and combining values 3-5 into 0 “Not afraid.”

#### Sociodemographic Information

In the baseline questionnaire participants self-reported gender (male; female; other), age at study (years), highest education achieved: primary (11 years of mandatory school); secondary (vocational, technical or high school) and tertiary (university or college degree) [20], and current monthly (gross) household income in Swiss Francs (Table 1). We categorized income into four categories: ≤6,000; 6,001 to ≤12,000; 12,001 to ≤18,000 and >18,001.

#### Health-Related Information

We coded the self-reported presence of chronic conditions (cancer, diabetes, immunocompromised, hypertension, CVD, chronic respiratory disease) into: none (no chronic condition); only one (listed for each condition separately) and more than one (more than one chronic condition).

### Data Analysis

Descriptive statistics are presented as frequencies and percentages for categorical variables and means with standard deviations (SD) for continuous variables (or medians and interquartile ranges for non-normal distributed variables). We compared the characteristics of the participants needing regular treatment and reporting a change in healthcare utilization at least once, versus those needing regular treatment but not changing their ongoing treatment with descriptive and bivariate statistics, including t-tests, χ^2^ tests, and Fisher’s exact tests (binary). Due to differing wording in the answer possibilities in the assessment of fear about potential COVID-19 infection as a possible reason, this data could not be harmonized across all research sites. This resulted in only two sites (BS and BL) being included in the present analyses. We compared fear between those who kept their ongoing treatment and those who attended an unscheduled consultation in each month using Fisher’s exact tests. The frequency of changes in those needing regular treatment showed an overdispersion in the Poisson regression. We therefore performed a multivariable zero-inflated negative binomial regression to evaluate risk factors associated with the rate of changes in healthcare utilization as a count variable.

The significance threshold was defined by a *p*-value ≤0.05. Statistical analyses were performed using STATA version 17 (StataCorp, College Station, TX, United States).

We performed a frequency analysis of the textual data to the answer possibility of other reasons for a change in utilization using ATLAS.ti Scientific Software Development GmbH [ATLAS.ti 22 Windows] (2022). The data has been visualized using the word cloud feature, where words used in a text are separated and the most frequently used words appear larger or bolder.

## Results

Out of 6,252 individuals of the eCohort in the nine centers, 3,190 adults (response rate: 51%) completed at least one monthly questionnaire assessing healthcare utilization from September 2020 to February 2021.

About one-fifth of the participants (*n* = 658, 21%, Figure 1) needed regular treatment. Among them, 14% (*n* = 90) reported at least one change in regular healthcare utilization during the observation period.

The mean age was 54 years (range: 20–95 years, Table 1). The majority were from the German-speaking region (81%), of Swiss nationality (80%), employed (full- or part-time, 58%), highly educated (tertiary education, 50%), and did not report having cancer, diabetes, hypertension, CVD, chronic respiratory disease or being immunocompromised as a chronic health condition, 70%.

The change in the incidence of healthcare utilization was one in 1,000 person-days. Those, who reported a change in healthcare utilization were significantly more likely female (*p* = 0.002, Table 1), non-Swiss (*p* = 0.03), and having only hypertension (*p* = 0.044).

**FIGURE 2:**
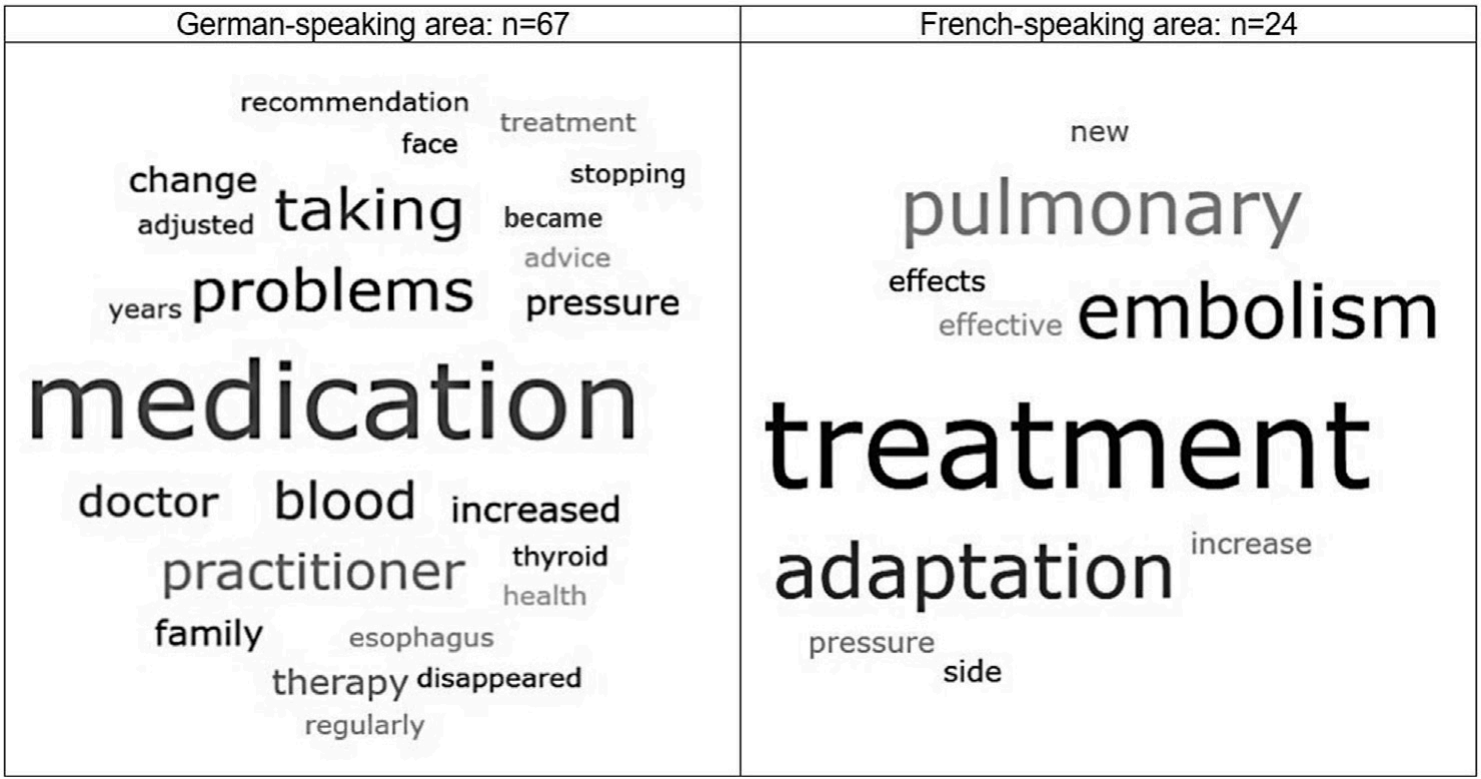
Reasons for change in healthcare utilization, Corona Immunitas eCohort of nine centers (Switzerland, September 2020–February 2021).

Reasons for changes in healthcare utilization in the German-speaking area (*n* = 67, 74%, Figure 2) were mostly attributed to adaptation/control of medication, specifically for blood pressure treatment. In contrast, in the French-speaking area (*n* = 24, 26%) these were mostly attributed to treatment for pulmonary embolism.

**FIGURE 3:**
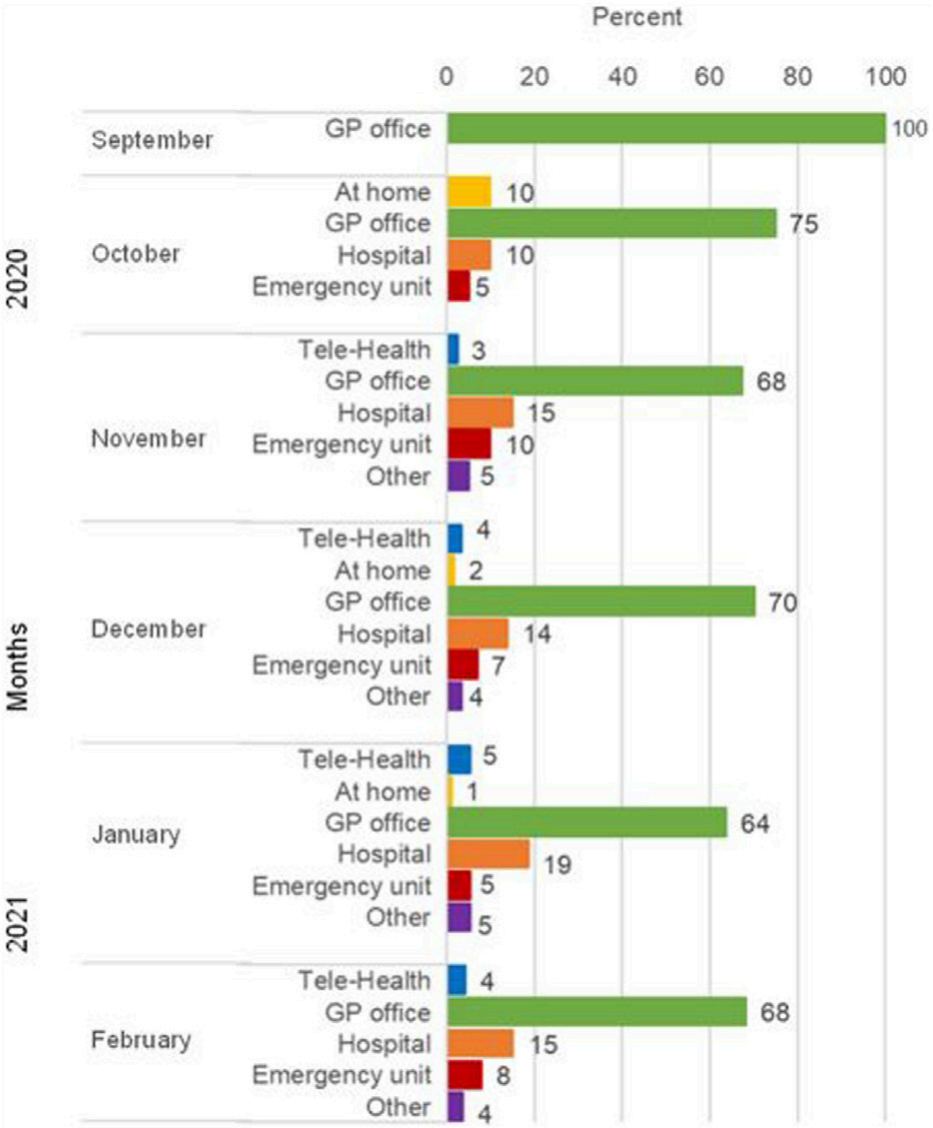
Type of consultation in those who did not need regular treatment (*n* = 345), eCohort of nine centers (Switzerland, September 2020–February 2021). Abbreviation: GP, general practice.

Among those who did not need regular treatment and had a consultation that was not COVID-related (*n* = 345, Figure 3), the majority (range 64%–100%) went to the GP office or attended a hospital (range 10%–19%). Telemedicine was used by a few participants (range 3%–5%), mainly in the winter months.

**FIGURE 4:**
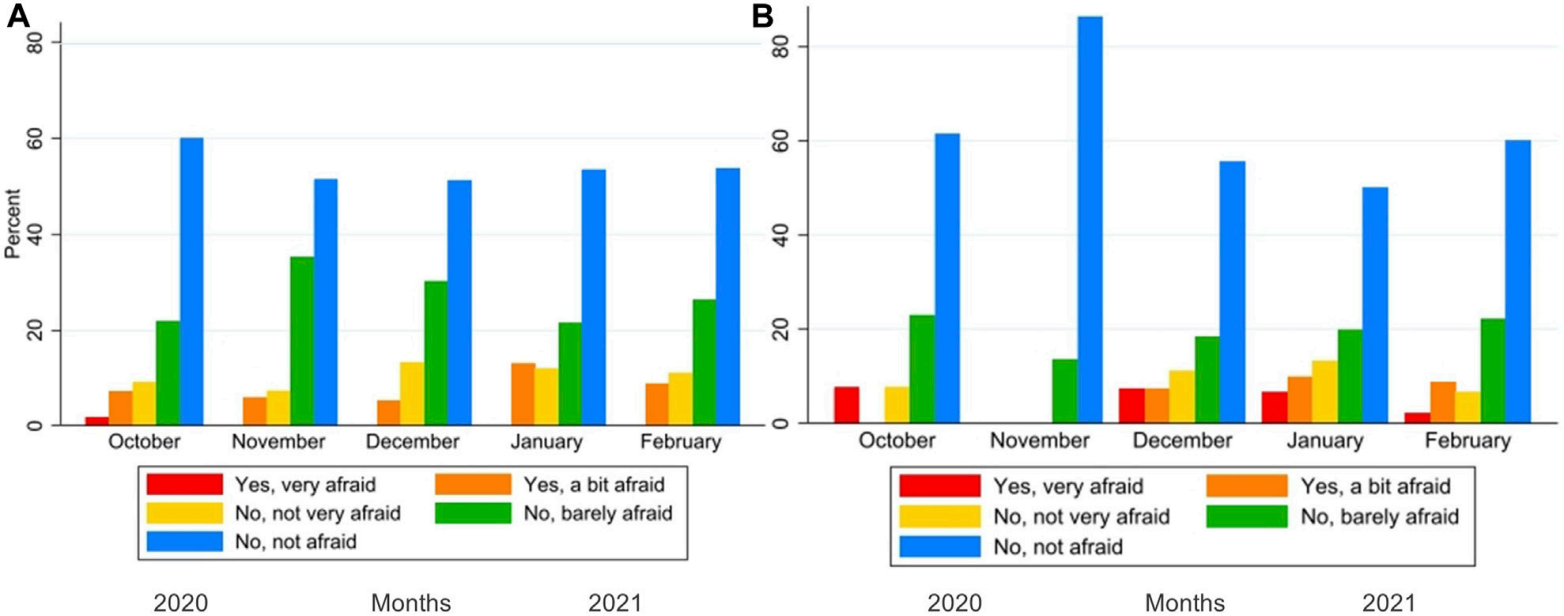
Fear of infection during regular treatment **(A)**, or during consultation **(B)**. Corona Immunitas eCohort of nine centers (Switzerland, September 2020–February 2021).

We found low fear of infection with COVID-19 in both groups, in those attending regular treatment and in those who had an unplanned consultation (Figure 4). In each month, fear did not differ between the two groups (Fisher’s exact tests, Figure 4).

**FIGURE 5:**
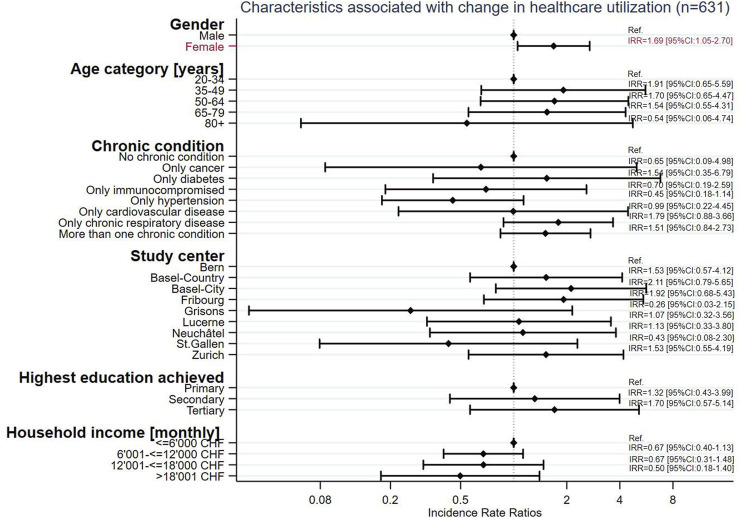
Characteristics associated with change in healthcare utilization (*n* = 631), Corona Immunitas eCohort of nine centers (Switzerland, September 2020–February 2021).

Females were significantly more likely to report changes in healthcare utilization (IRR = 1.69, 95% CI: 1.05–2.70, *p* = 0.030) and those with hypertension were least likely to report a change (IRR = 0.45, 95% CI: 0.18–1.14, *p* = 0.092) as compared to those with other chronic conditions (Figure 5).

## Discussion

From September 2020 to February 2021, 14% of those who needed ongoing treatment reported a change in healthcare utilization, corresponding to one in 1,000 persons per day. We found that overall, the fear of contracting COVID-19 was low during regular treatment and non-COVID-19-related unplanned consultations. Reasons for change in healthcare utilization were reported as mainly medication-related in the German-speaking area, especially for blood pressure treatment and side effects. In contrast, treatment for pulmonary embolism was the main reason in the French-speaking area. Most participants attended the unplanned consultations at the GP office or a hospital and few used telemedicine. Females were more likely to report changes in healthcare utilization, and those with hypertension were least likely to report changes.

In contrast to other studies worldwide in different healthcare systems and settings, we found relatively low rates of change in regular treatment [1, 4]. A systematic review on general health concerns included 81 studies from 20 countries that reported a consistent decline in health service utilization early during the pandemic through to August 2020, compared to previous years [1]. In 45% of the studies relating to change according to disease severity, the systematic review found a greater decrease in healthcare utilization among people with less severe illnesses. Studies also reported increasing health service utilization related to telemedicine [9, 10]. Findings included different healthcare systems and settings.

Our findings are in line with another Swiss study where in the year 2020 expected values returned soon after the end of the lockdown [6]. Another study from Switzerland also found similar reductions in healthcare utilization between the general population and at-risk patients [4]. In this last study, the authors attributed the decrease in healthcare utilization to governmental restrictions such as the closing of non-essential businesses and schools, and even a ban on non-urgent healthcare services imposed in the early months of 2020 [4, 8]. A study of cancer patients showed delays and interruptions in healthcare primarily related to reductions in available health services during the pandemic [21]. During our study period, restrictions in Switzerland varied. Some restrictions had already been lifted assuming a return to normalcy, while others imposed cantonal restrictions. Vaud, Valais, Ticino, and Zurich were among the ten cantons with higher stringency compared to the national measures [22]. Additionally, we found that the fear of contracting COVID-19 was low when seeking medical care. This finding contrasts with other studies where concerns about cross-infection and beliefs of individual vulnerability contributed to a decline in healthcare utilization [5, 23–25]. One reason for the general low fear in this study may be that the initial fear and COVID-19-related insecurities may have subsided given that people had gathered knowledge on how to protect themselves.

Participants reported changes in healthcare utilization due to medication, particularly for blood pressure treatment, side effects, and pulmonary embolism. This may be related to the fact, that among self-reported chronic conditions, hypertension was the most common chronic condition in our study sample. A recent study in Turkey found that the available information and understanding of the effects of COVID-19 positively influenced treatment adherence and whether participants had their blood pressure controlled during the pandemic [26]. This could either result in an increase or maintenance of regular healthcare utilization. Hypertension requires regular monitoring, which, after careful and empowering instruction, can be performed independently at home. A recent study from Ethiopia found that uncontrolled blood pressure was negatively associated with low education and poor medication adherence during the pandemic [27]. In our study, about half of the participants had achieved tertiary education. We found no association between educational achievement and changes in healthcare utilization. Yet, we found that participants with hypertension were less likely to change their ongoing treatment compared to others. Those experiencing difficulties with their medication and with better health literacy may have been more likely to engage in appropriate measures and contacted their healthcare providers in a timely manner.

Some studies have reported increased health service utilization related to telemedicine [9, 10]. In Switzerland, telemedicine was offered as an option for the upkeep of healthcare provision. However, in our study, it was used only by a few individuals during the study period. One reason could be that individuals did not perceive the need for telemedicine, as fear of infection was low, and in-person consultations had resumed. Another reason could be that telemedicine may not have been available everywhere.

People with higher education had a slightly higher change rate, while people with higher income were less likely to report changes in healthcare utilization. This could be explained by the different assessments in the study. Highest achieved education was self-reported by the individual study participant as a personal characteristic, whereas monthly household income was reported on the household level the individual lives in. Here, the reported income may not be the individuals own income. About half of the participants had achieved tertiary education. Most participants however, reported a monthly household income, ranging from 6,001 to ≤12,000 CHF/month. Additionally, the proportion of retired individuals with tertiary education could have contributed to this contradiction, as they may have achieved a high education but currently have a low monthly retirement income. In our study, women were more likely to report changes in healthcare utilization. Because schools had resumed operating, pre-pandemic arrangements made for childcare would have resumed as well. Explanations for changes in healthcare utilization in women other than childcare must be explored. A recent study involving eight high-income countries found a large gender difference in the perceived severity of COVID-19 as a health risk. More women than men considered the disease a serious health problem and agreed to government-imposed restrictions, such as self-quarantine at home, and closing non-essential economic activities and facilities [28]. Furthermore, compliance with public health and social distancing measures (e.g., washing hands more often, changing greeting habits, avoiding crowded places, and stopping visits to friends) was higher among women than men [28]. These results were reported in the early months of 2020. When we conducted our study, the initial shock of the pandemic may have subsided even though the pandemic was not over. Additionally, unhealthy behaviors related to recommended homestays such as unhealthy diet, reduced physical activity, and mental health problems may have also influenced healthcare utilization and exacerbated long-term health consequences [29].

Lessons from this pandemic may include identifying (un)needed care in health systems and implementing new solutions to maintain essential routine monitoring to achieve greater sustainability in post-pandemic recovery [1, 30].

### Strengths and Limitations

The main strength of this analysis is the longitudinal data from the Swiss general population collected monthly over 6 months through standardized questionnaires. Another strength is the digital data collection permitting participation from anywhere and at any time. The participation rate is in line with other survey-based studies. Despite the digital design, we report the successful participation of many individuals older than 65 years. Our study included representative samples in both age groups (20–64 years and 65+ years) within the Swiss general population. Unfortunately, the data on fear about potential COVID-19 infection could not be harmonized and only two out of nine research sites were included in this analysis. However, we expect that reports of fear in Switzerland would have been similar in all centers. As in other studies, selection bias cannot be ruled out, as the majority did not report a chronic condition and were highly educated. The relatively low response rate is another limitation. Individuals with more severe conditions and health needs may therefore be underrepresented in this study’s findings. We did not perform a non-participant analysis due to a lack of information on these factors among non-participants.

### Conclusion

Our study contributes to understanding changes in healthcare utilization in the general population in Switzerland during the COVID-19 pandemic. Changes in healthcare utilization were reported by few persons who needed regular treatment, corresponding to an incidence of one in 1’000 person-days. The importance of continuity of care for chronic diseases other than hypertension must be emphasized. Careful and encouraging instructions for self-management may be helpful. Changes were more pronounced in women than in men. This calls for tailored disease surveillance, considering gender disparities.

Previously reported disruptions in chronic disease surveillance make it imperative for future studies to assess the long-term impact of healthcare utilization during the pandemic on health outcomes.

## Data Availability

Data is available on request by contacting Corona Immunitas.

## Corona Immunitas Research Group

Emiliano Albanese (Institute of Public Health (IPH), Università della Svizzera italiana, Lugano, Switzerland); Rebecca Amati (Institute of Public Health (IPH), Università della Svizzera italiana, Lugano, Switzerland); Antonio Amendola (Department of Business Economics, Health and Social Care (DEASS), University of Applied Sciences & Arts of Southern Switzerland (SUPSI), Switzerland); Alexia Anagnostopoulos (Epidemiology, Biostatistics and Prevention Institute, University of Zurich, Zurich, Switzerland); Daniela Anker (Population Health Laboratory (#PopHealthLab), University of Fribourg, Switzerland; Institute of Primary Health Care (BIHAM), University of Bern, Switzerland); Anna Maria Annoni (Institute of Public Health (IPH), Università della Svizzera italiana, Lugano, Switzerland); Hélène Aschmann (Epidemiology, Biostatistics and Prevention Institute, University of Zurich, Zurich, Switzerland); Andrew Azman (Unit of Population Epidemiology, Division of Primary Care Medicine, Geneva University Hospitals, Geneva, Switzerland; Department of Epidemiology, Johns Hopkins Bloomberg School of Public Health, Baltimore, MD, USA; Institute of Global Health, Faculty of Medicine, University of Geneva, Geneva, Switzerland); Antoine Bal (Unit of Population Epidemiology, Division of Primary Care Medicine, Geneva University Hospitals, Geneva, Switzerland); Tala Ballouz (Epidemiology, Biostatistics and Prevention Institute, University of Zurich, Zurich, Switzerland); Hélène Baysson (Unit of Population Epidemiology, Division of Primary Care Medicine, Geneva University Hospitals, Geneva, Switzerland; Department of Health and Community Medicine, Faculty of Medicine, University of Geneva, Geneva, Switzerland); Kleona Bezani (Institute of Public Health (IPH), Università della Svizzera italiana, Lugano, Switzerland); Annette Blattmann (Cantonal Hospital St. Gallen, Clinic for Infectious Diseases and Hospital Epidemiology, St. Gallen, Switzerland); Patrick Bleich (Unit of Population Epidemiology, Division of Primary Care Medicine, Geneva University Hospitals, Geneva, Switzerland); Murielle Bochud (Center for Primary Care and Public Health (Unisanté), University of Lausanne, Switzerland); Patrick Bodenmann (Center for Primary Care and Public Health (Unisanté), University of Lausanne, Switzerland); Gaëlle Bryand Rumley (Unit of Population Epidemiology, Division of Primary Care Medicine, Geneva University Hospitals, Geneva, Switzerland); Peter Buttaroni (Institute of Public Health (IPH), Università della Svizzera italiana, Lugano, Switzerland); Audrey Butty (Center for Primary Care and Public Health (Unisanté), University of Lausanne, Switzerland); Anne Linda Camerini (Institute of Public Health (IPH), Università della Svizzera italiana, Lugano, Switzerland); Arnaud Chiolero (Population Health Laboratory (#PopHealthLab), University of Fribourg, Switzerland; Institute of Primary Health Care (BIHAM), University of Bern, Switzerland; Department of Epidemiology, Biostatistics and Occupational Health, McGill University, Montréal, Canada); Patricia Orializ Chocano-Bedoya (Institute of Primary Health Care (BIHAM), University of Bern; Population Health Laboratory (#PopHealthLab), University of Fribourg, Switzerland); Prune Collombet (Unit of Population Epidemiology, Division of Primary Care Medicine, Geneva University Hospitals, Geneva, Switzerland; Department of Health and Community Medicine, Faculty of Medicine, University of Geneva, Geneva, Switzerland); Laurie Corna (Department of Business Economics, Health and Social Care (DEASS), University of Applied Sciences & Arts of Southern Switzerland (SUPSI), Switzerland); Luca Crivelli (Department of Business Economics, Health and Social Care (DEASS), University of Applied Sciences & Arts of Southern Switzerland (SUPSI), Switzerland; Institute of Public Health (IPH), Università della Svizzera italiana, Lugano, Switzerland); Stéphane Cullati (Population Health Laboratory (#PopHealthLab), University of Fribourg, Switzerland; Department of Readaptation and Geriatrics, University of Geneva, Switzerland); Valérie D'Acremont (Center for Primary Care and Public Health (Unisanté), University of Lausanne, Switzerland; Swiss Tropical and Public Health Institute, Basel, Switzerland); Diana Sofia Da Costa Santos (Institute of Public Health (IPH), Università della Svizzera italiana, Lugano, Switzerland); Agathe Deschamps (Cantonal Medical Service Neuchâtel); Paola D’Ippolito (Unit of Population Epidemiology, Division of Primary Care Medicine, Geneva University Hospitals, Geneva, Switzerland); Anja Domenghino (Epidemiology, Biostatistics and Prevention Institute, University of Zurich, Zurich, Switzerland); Richard Dubos (Unit of Population Epidemiology, Division of Primary Care Medicine, Geneva University Hospitals, Geneva, Switzerland); Roxane Dumont (Unit of Population Epidemiology, Division of Primary Care Medicine, Geneva University Hospitals, Geneva, Switzerland); Olivier Duperrex (Center for Primary Care and Public Health (Unisanté), University of Lausanne, Switzerland); Julien Dupraz (Center for Primary Care and Public Health (Unisanté), University of Lausanne, Switzerland); Malik Egger (Center for Primary Care and Public Health (Unisanté), University of Lausanne, Switzerland); Emna El-May (Population Health Laboratory (#PopHealthLab), University of Fribourg, Switzerland); Nacira El Merjani (Unit of Population Epidemiology, Division of Primary Care Medicine, Geneva University Hospitals, Geneva, Switzerland); Nathalie Engler (Cantonal Hospital St. Gallen, Clinic for Infectious Diseases and Hospital Epidemiology, St. Gallen, Switzerland); Adina Mihaela Epure (Population Health Laboratory (#PopHealthLab), University of Fribourg, Switzerland); Lukas Erksam (Institute of Primary Health Care (BIHAM), University of Bern, Department of General Internal Medicine, Inselspital, Bern University Hospital, University of Bern); Sandrine Estoppey (Center for Primary Care and Public Health (Unisanté), University of Lausanne, Switzerland); Marta Fadda (Institute of Public Health (IPH), Università della Svizzera italiana, Lugano, Switzerland); Vincent Faivre (Center for Primary Care and Public Health (Unisanté), University of Lausanne, Switzerland); Jan Fehr (Epidemiology, Biostatistics and Prevention Institute, University of Zurich, Zurich, Switzerland); Andrea Felappi (Center for Primary Care and Public Health (Unisanté), University of Lausanne, Switzerland); Maddalena Fiordelli (Institute of Public Health (IPH), Università della Svizzera italiana, Lugano, Switzerland); Antoine Flahault (Institute of Global Health, Faculty of Medicine, University of Geneva, Geneva, Switzerland; Division of Tropical and Humanitarian Medicine, Geneva University Hospitals, Geneva, Switzerland; Department of Health and Community Medicine, Faculty of Medicine, University of Geneva, Geneva, Switzerland); Luc Fornerod (Observatoire valaisan de la santé (OVS), Sion, Switzerland); Cristina Fragoso Corti (Department of environment construction and design (DACD, University of Applied Sciences & Arts of Southern Switzerland (SUPSI), Switzerland); Natalie Francioli (Unit of Population Epidemiology, Division of Primary Care Medicine, Geneva University Hospitals, Geneva, Switzerland); Marion Frangville (Unit of Population Epidemiology, Division of Primary Care Medicine, Geneva University Hospitals, Geneva, Switzerland); Irène Frank, (Luzerner Kantonsspital, Spitalstrasse, 6000 Luzern 16); Giovanni Franscella (Institute of Public Health (IPH), Università della Svizzera italiana, Lugano, Switzerland); Anja Frei (Epidemiology, Biostatistics and Prevention Institute, University of Zurich, Zurich, Switzerland); Marco Geigges (Epidemiology, Biostatistics and Prevention Institute, University of Zurich, Zurich, Switzerland); Semira Gonseth Nusslé (Center for Primary Care and Public Health (Unisanté), University of Lausanne, Switzerland); Clément Graindorge (Unit of Population Epidemiology, Division of Primary Care Medicine, Geneva University Hospitals, Geneva, Switzerland); Idris Guessous (Unit of Population Epidemiology, Division of Primary Care Medicine, Geneva University Hospitals, Geneva, Switzerland; Department of Health and Community Medicine, Faculty of Medicine, University of Geneva, Geneva, Switzerland); Erika Harju (Faculty of Health Sciences and Medicine, University of Lucerne, Lucerne); Séverine Harnal (Unit of Population Epidemiology, Division of Primary Care Medicine, Geneva University Hospitals, Geneva, Switzerland); Medea Imboden (Swiss Tropical and Public Health Institute, Department of Epidemiology and Public Health, Basel, Switzerland; University of Basel, Basel, Switzerland); Emilie Jendly (Center for Primary Care and Public Health (Unisanté), University of Lausanne, Switzerland); Ayoung Jeong (Swiss Tropical and Public Health Institute, Department of Epidemiology and Public Health, Basel, Switzerland; University of Basel, Basel, Switzerland); Christian R Kahlert (Cantonal Hospital St. Gallen, Clinic for Infectious Diseases and Hospital Epidemiology, St. Gallen, Switzerland; Children's Hospital of Eastern Switzerland, Infectious Diseases and Hospital Epidemiology, St. Gallen, Switzerland); Laurent Kaiser (Geneva Center for Emerging Viral Diseases and Laboratory of Virology, Geneva University Hospitals, Geneva, Switzerland; Division of Infectious Diseases, Geneva University Hospitals, Geneva, Switzerland; Department of Medicine, Faculty of Medicine, University of Geneva, Geneva, Switzerland); Laurent Kaufmann (Service de La Santé Publique, Canton de Neuchâtel, Neuchâtel, Switzerland); Marco Kaufmann (Epidemiology, Biostatistics and Prevention Institute, University of Zurich, Zurich, Switzerland); Dirk Keidel (Swiss Tropical and Public Health Institute, Department of Epidemiology and Public Health, Basel, Switzerland; University of Basel, Basel, Switzerland); Simone Kessler (Cantonal Hospital St. Gallen, Clinic for Infectious Diseases and Hospital Epidemiology, St. Gallen, Switzerland); Philipp Kohler (Cantonal Hospital St. Gallen, Clinic for Infectious Diseases and Hospital Epidemiology, St. Gallen, Switzerland); Christine Krähenbühl (Luzerner Kantonsspital, Spitalstrasse, 6000 Luzern 16); Susi Kriemler (Epidemiology, Biostatistics and Prevention Institute, University of Zurich, Zurich, Switzerland); Julien Lamour (Unit of Population Epidemiology, Division of Primary Care Medicine, Geneva University Hospitals, Geneva, Switzerland); Sara Levati (Department of Business Economics, Health and Social Care (DEASS), University of Applied Sciences & Arts of Southern Switzerland (SUPSI), Switzerland); Pierre Lescuyer (Division of Laboratory Medicine, Geneva University Hospitals, Geneva, Switzerland); Andrea Loizeau (Unit of Population Epidemiology, Division of Primary Care Medicine, Geneva University Hospitals, Geneva, Switzerland); Elsa Lorthe (Unit of Population Epidemiology, Division of Primary Care Medicine, Geneva University Hospitals, Geneva, Switzerland); Chantal Luedi (Faculty of Health Sciences and Medicine, University of Lucerne, Lucerne); Jean-Luc Magnin, PhD (Laboratory, HFR-Fribourg, Fribourg, Switzerland); Chantal Martinez (Unit of Population Epidemiology, Division of Primary Care Medicine, Geneva University Hospitals, Geneva, Switzerland); Eric Masserey (Cantonal Medical Office, General Health Department, Canton of Vaud, Switzerland); Dominik Menges (Epidemiology, Biostatistics and Prevention Institute, University of Zurich, Zurich, Switzerland); Gisela Michel (Faculty of Health Sciences and Medicine, University of Lucerne, Lucerne); Rosalba Morese (Faculty of Communication, Culture and Society, Università della Svizzera italiana, Lugano, Switzerland; Faculty of Biomedical Sciences, Università della Svizzera italiana, Lugano, Switzerland); Nicolai Mösli (Swiss TPH, Basel, Switzerland; University of Basel, Basel, Swtizerland); Natacha Noël (Unit of Population Epidemiology, Division of Primary Care Medicine, Geneva University Hospitals, Geneva, Switzerland); Daniel Henry Paris (Swiss TPH, Basel, Switzerland; University of Basel, Basel, Swtizerland); Jérôme Pasquier (Center for Primary Care and Public Health (Unisanté), University of Lausanne, Switzerland); Francesco Pennacchio (Unit of Population Epidemiology, Division of Primary Care Medicine, Geneva University Hospitals, Geneva, Switzerland); Stefan Pfister (Laboratory, HFR-Fribourg, Fribourg, Switzerland); Giovanni Piumatti (Fondazione Agnelli, Turin, Italy); Géraldine Poulain (Division of Laboratory Medicine, Geneva University Hospitals, Geneva, Switzerland); Nicole Probst-Hensch (Swiss Tropical and Public Health Institute, Department of Epidemiology and Public Health, Basel, Switzerland; University of Basel, Basel, Swtizerland); Caroline Pugin (Unit of Population Epidemiology, Division of Primary Care Medicine, Geneva University Hospitals, Geneva, Switzerland); Milo A. Puhan, (Epidemiology, Biostatistics and Prevention Institute, University of Zurich, Zurich, Switzerland); Nick Pullen (Unit of Population Epidemiology, Division of Primary Care Medicine, Geneva University Hospitals, Geneva, Switzerland); Thomas Radtke (Epidemiology, Biostatistics and Prevention Institute, University of Zurich, Zurich, Switzerland); Manuela Rasi (Epidemiology, Biostatistics and Prevention Institute, University of Zurich, Zurich, Switzerland); Aude Richard (Unit of Population Epidemiology, Division of Primary Care Medicine, Geneva University Hospitals, Geneva, Switzerland; Institute of Global Health, University of Geneva, Switzerland); Viviane Richard (Unit of Population Epidemiology, Division of Primary Care Medicine, Geneva University Hospitals, Geneva, Switzerland); Claude-François Robert (Cantonal Medical Service Neuchâtel); Pierre-Yves Rodondi (Institute of Family Medicine, University of Fribourg, Fribourg, Switzerland); Nicolas Rodondi (Institute of Primary Health Care (BIHAM), University of Bern; Department of General Internal Medicine, Inselspital, Bern University Hospital, University of Bern); Serena Sabatini (Institute of Public Health (IPH), Università della Svizzera italiana, Lugano, Switzerland); Khadija Samir (Unit of Population Epidemiology, Division of Primary Care Medicine, Geneva University Hospitals, Geneva, Switzerland); Javier Sanchis Zozaya (Center for Primary Care and Public Health (Unisanté), University of Lausanne, Switzerland); Virginie Schlüter (Center for Primary Care and Public Health (Unisanté), University of Lausanne, Switzerland); Alexia Schmid (Institute of Family Medicine, University of Fribourg, Fribourg, Switzerland); Valentine Schneider (Cantonal Medical Service Neuchâtel); Maria Schüpbach (Institute of Primary Health Care (BIHAM), University of Bern, Department of General Internal Medicine, Inselspital, Bern University Hospital, University of Bern); Nathalie Schwab (Institute of Primary Health Care (BIHAM), University of Bern, Department of General Internal Medicine, Inselspital, Bern University Hospital, University of Bern); Claire Semaani (Unit of Population Epidemiology, Division of Primary Care Medicine, Geneva University Hospitals, Geneva, Switzerland); Alexandre Speierer (Institute of Primary Health Care (BIHAM), University of Bern; Department of General Internal Medicine, Inselspital, Bern University Hospital, University of Bern); Amélie Steiner-Dubuis (Center for Primary Care and Public Health (Unisanté), University of Lausanne, Switzerland); Silvia Stringhini (Unit of Population Epidemiology, Division of Primary Care Medicine, Geneva University Hospitals, Geneva, Switzerland; Department of Health and Community Medicine, Faculty of Medicine, University of Geneva, Geneva, Switzerland); Stefano Tancredi (Population Health Laboratory (#PopHealthLab), University of Fribourg, Switzerland); Stéphanie Testini (Unit of Population Epidemiology, Division of Primary Care Medicine, Geneva University Hospitals, Geneva, Switzerland); Julien Thabard (Center for Primary Care and Public Health (Unisanté), University of Lausanne, Switzerland); Mauro Tonolla (Department of environment construction and design (DACD, University of Applied Sciences & Arts of Southern Switzerland (SUPSI), Switzerland); Nicolas Troillet (Office du médecin cantonal, Sion, Switzerland); Agne Ulyte (Epidemiology, Biostatistics and Prevention Institute, University of Zurich, Zurich, Switzerland); Sophie Vassaux (Center for Primary Care and Public Health (Unisanté), University of Lausanne, Switzerland); Thomas Vermes (Swiss Tropical and Public Health Institute, Department of Epidemiology and Public Health, Basel, Switzerland; University of Basel, Basel, Swtizerland); Jennifer Villers (Unit of Population Epidemiology, Division of Primary Care Medicine, Geneva University Hospitals, Geneva, Switzerland); Viktor von Wyl (Epidemiology, Biostatistics and Prevention Institute, University of Zurich, Zurich, Switzerland); Cornelia Wagner (Population Health Laboratory (#PopHealthLab), University of Fribourg, Switzerland); Rylana Wenger (Institute of Primary Health Care (BIHAM), University of Bern, Department of General Internal Medicine, Inselspital, Bern University Hospital, University of Bern); Erin West (Epidemiology, Biostatistics and Prevention Institute, University of Zurich, Zurich, Switzerland); Ania Wisniak (Unit of Population Epidemiology, Division of Primary Care Medicine, Geneva University Hospitals, Geneva, Switzerland; Institute of Global Health, Faculty of Medicine, University of Geneva, Geneva, Switzerland); Melissa Witzig (Swiss Tropical and Public Health Institute, Department of Epidemiology and Public Health, Basel, Switzerland; University of Basel, Basel, Swtizerland); María-Eugenia Zaballa (Unit of Population Epidemiology, Division of Primary Care Medicine, Geneva University Hospitals, Geneva, Switzerland); Kyra Zens (Epidemiology, Biostatistics and Prevention Institute, University of Zurich, Zurich, Switzerland); Claire Zuppinger (Center for Primary Care and Public Health (Unisanté), University of Lausanne, Switzerland).
