# Supplementary material for: Changes in Healthcare Utilization During the COVID-19 Pandemic and Potential Causes—A Cohort Study From Switzerland
Source: Int J Public Health. 2023 Jul 26;68:1606010. doi: 10.3389/ijph.2023.1606010 (PMC10469983; doi:10.3389/ijph.2023.1606010)
Supplement: Supplementary file 1 [file DataSheet1.docx]

**Supplementary material: Monthly Questionnaire**

| **Question** | | **Answers** |
| --- | --- | --- |
| Over the past 7 days, have you had any new  symptoms unrelated to pre-existing chronic illness or allergies? | | □ No; □ Yes |
| During the last 7 days, have you had the following symptoms (new symptoms unrelated to preexisting  chronic illness or allergies)? For each symptom, state yes or no): | |  |
| Dry cough | | □ No; □ Yes |
| Feeling feverish | | □ No; □ Yes |
| Cough with mucus | | □ No; □ Yes |
| Body temperature of 38°C (100.4°F) or more | | □ No; □ Yes |
| Coughing or spitting blood | | □ No; □ Yes |
| Runny or stuffy nose | | □ No; □ Yes |
| Sneezing | | □ No; □ Yes |
| Sore throat | | □ No; □ Yes |
| Breathlessness | | □ No; □ Yes |
| Difficulty breathing | | □ No; □ Yes |
| Headache | | □ No; □ Yes |
| Muscle and / or joint pain | | □ No; □ Yes |
| Chest, thorax, and / or sternum pain | | □ No; □ Yes |
| Fatigue or exhaustion | | □ No; □ Yes |
| Loss of appetite | | □ No; □ Yes |
| Nausea and / or vomiting | | □ No; □ Yes |
| Diarrhea | | □ No; □ Yes |
| Stomachache | | □ No; □ Yes |
| Loss of smell and / or taste | | □ No; □ Yes |
| Irritated and / or watery eyes | | □ No; □ Yes |
| Skin rash | | □ No; □ Yes |
| Other symptoms | | □ No; □ Yes |
| Other symptoms (please specify) | | ______________________________________ |
| When the first symptoms appeared, what did you do? (You can select multiple answers) | | □ You called your doctor; □ You went to the doctor's office; □ You went to a hospital emergency room; □ You called a coronavirus medico-sanitary hotline of the Service du médecin cantonal; □ You called emergency services (144); □ You called a medical counselling service (e.g., insurance); □ You went to the pharmacy; □ You stayed home; □ Other; □ You got tested for the Coronavirus; □ No particular actions taken |
| Other (please specify) | | ______________________________________ |
| Over the past 7 days, have you taken any medicine to treat those new symptoms? (You may select multiple answers) | | □ No medicine; □ Paracetamol (Dafalgan; NeoCitran, Pretuval, Panadol, Ben-U-RON, Zolben, etc.); □ Cortisone, Prednisone; □ Nonsteroidal anti- inflammatory (Ibuprofen, Irfen, Algifor, Brufen, etc.); □ Other analgesic / anti-inflammatory: aspirin (Aspegic), naproxen, diclofenac; □ Antiviral drugs (Tamiflu, ritonavir, lopinavir); □ Antibiotics (Amoxicilline, Azithromycin, Bactrim); □ Spray (Atrovent, Bricanyl, Dospir, Seretide, Ventolin, Symbicort, Spiriva, etc.) for respiratory problems (asthma or chronic bronchitis); □ Hydroxychloroquine (Plaquenil); □ Other |
| Other (please specify) | | ______________________________________ |
| Over the past 7 days, have you been hospitalized because of the symptoms you're reporting today? | | □ No; □ Yes |
| If yes, how long did the hospitalisation last? (Number) | | ______________________________________ |
| Date of start | | ______________________________________ |
| Date of end | | ______________________________________ |
| I'm still hospitalised | | □ No; □ Yes |
| During this hospitalisation, have you been admitted to | | □ No; □ Yes |
| If yes, how many days? | | ______________________________________ |
| Over the past 7 days, have you been tested for  coronavirus (SARS-CoV-2)? (nasal or oral swab, saliva test) | | □ No; □ Yes, once; □ I do not know / I do not want to answer this question; □ Yes, many times |
| If yes (once), when exactly? | | ______________________________________ |
| The result of the test was | | □ Positive (virus present); □ Negative (virus not present); □ Test result still pending; □ I do not know / I do not want to answer this question |
| Who performed the test? | | □ Health-care professional; □ Yourself; □ Someone close to you (e.g.  family member, friend) |
| Which type of test was used? | | □ PCR test (test sent to a lab, result received within 24-48 hours);  □ Antigen rapid test (test result within 15-30 minutes); □ I don't know; □ A covid self-test (to be done at home) |
| If yes (many times), have you ever received a positive result (virus present)? | | □ No (the results were negative each time - absence of the virus); □ Yes, I had a positive result |
| When exactly did you receive the last positive result? | | ______________________________________ |
| Who performed the test? | | □ Health-care professional; □ Yourself; □ Someone close to you (e.g.  family member, friend) |
| Which type of test was used? | | □PCR test (test sent to a lab, result received within 24-48 hours); □Antigen rapid test (test result within 15-30 minutes); □I don't know; □A covid self-test (to be done at home) |
| Over the past 7 days, have you been medically  followed-up at home by a coronavirus medical follow-up (provided by your canton)? | | □ No; □ Yes |
| Over the past 7 days, have you done a blood test  (serology) to detect if you had antibodies against SARS-CoV-2? (other than Corona-Immunitas study) | | □No; □Yes; □I don't know / I do not want to answer |
| At what date? | | ______________________________________ |
| The results of the test were | | □Positive (presence of antibodies); □Negative (absence of antibodies); □Waiting for results; □I don't know / I don't want to answer |
| Over the past 7 days, how frequently have you ... | |  |
| ... applied the recommended hygiene measures  (washing hands regularly, sneezing into the elbow, using disposable tissues, etc.)? | | □Never; □Very rarely; □Occasionally; □Frequently; □Constantly |
| ...implemented the recommended measures for social distancing (no handshaking or hugging, keep the distance of 1.5meters, etc.)? | | □Never; □Very rare; □Occasionally; □Frequently; □Always |
| ... applied the recommendations for staying at home (staying at home as much as possible, avoiding unnecessary outings or trips, etc.)? | | □Never; □Very rarely; □Occasionally; □Frequently; □Constantly |
| ... worn a mask due to the COVID-19 pandemic? | | □Never; □Very rare; □Occasionally; □Frequently; □Always |
| ...avoided non-essential in-person gatherings with persons outside of your own household? | | □Never; □Very rare; □Occasionally; □Frequently; □Always |
| In the last 7 days, do you think the risk of being  infected with COVID-19 is... (If you do not know, please press I do not know below) | | 0 Not at all, …, 100 Very high |
|  | | □I do not know |
| For the past 7 days, have you been forced to stay at home because of exposure to coronavirus (SARS-CoV- 2)? | | □Yes, because I have tested positive for coronavirus and / or have symptoms; □Yes, because I was in contact with a person who tested positive for coronavirus; □Yes, because a household member has tested positive for coronavirus or has symptoms; □Yes, because I am at risk (because of my age or a previous illness); □Yes, for another reason; □ No; □I don't know / don't want to answer ; □Yes, because I received a SwissCovid app notification that I was exposed; □ Yes, because I have spent more than 24 hours in an area listed by the federal office of public health for mandatory quarantine |
| If yes, please specify | |  |
| If you stayed at home (or are still at home), who asked you to do so? | | □I have followed the prescription of my doctor or the health specialist following me.; □I have followed the health recommendations currently applied in my canton.; □I have followed the recommendations of my employer or my occupational physician.; □I followed the recommendations of my relatives (friends, family); □I decided to stay  home by myself; □Other; □I have followed the order of the cantonal physician.; □ I stayed home because of a SwissCovid app notification |
| If other, specify | | ______________________________________ |
| How worried are you about the current corona-virus situation in the following areas: | |  |
| The consequences for my health | | □Not at all; □ A little; □Moderate; □Very; □Extreme |
| The health of relatives and friends | | □Not at all; □ A little; □Moderate; □Very; □Extreme |
| The risk of exposing myself to the virus | | □Not at all; □ A little; □Moderate; □Very; □Extreme |
| The risk of spreading the virus | | □Not at all; □ A little; □Moderate; □Very; □Extreme |
| My own economic and / or professional situation | | □Not at all; □ A little; □Moderate; □Very; □Extreme |
| The economic and / or professional situation of relatives and friends | | □Not at all; □ A little; □Moderate; □Very; □Extreme |
| The general economic situation in Switzerland | | □Not at all; □ A little; □Moderate; □Very; □Extreme |
| The quality of my family relationships | | □Not at all; □ A little; □Moderate; □Very; □Extreme |
| The quality of my personal relationships (e.g. with friends and colleagues) | | □Not at all; □ A little; □Moderate; □Very; □Extreme |
| The freedom of the Swiss population | | □Not at all; □ A little; □Moderate; □Very; □Extreme |
| The privacy of the Swiss population | | □Not at all; □ A little; □Moderate; □Very; □Extreme |
| To which extent do you think the following people are  worried about the current Coronavirus (SARS-CoV-2) situation: | |  |
| My family members | | □Not at all; □A little bit; □Moderately; □Much; □Extremely |
| Other people around me (e.g., friends neighbors, colleagues) | | □Not at all; □A little bit; □Moderately; □Much; □Extremely |
| Public health authorities | | □Not at all; □A little bit; □Moderately; □Much; □Extremely |
| News media | | □Not at all; □A little bit; □Moderately; □Much; □Extremely |
| People on social media | | □Not at all; □A little bit; □Moderately; □Much; □Extremely |
| Healthcare practitioners | | □Not at all; □A little bit; □Moderately; □Much; □Extremely |
| Politicians | | □Not at all; □A little bit; □Moderately; □Much; □Extremely |
| During the past 7 days, how often did you feel that you lack companionship? | | □Never; □Rarely; □Occasionally; □Most of the time; □Always |
| During the past 7 days, how often did you feel left out? | | □Never; □Rarely; □Occasionally; □Most of the time; □Always |
| During the past 7 days, how often did you feel isolated from others? | | □Never; □Rarely; □Occasionally; □Most of the time; □Always |
| Did you need medical treatment because of an ongoing illness during the past month? | | □ No; □ Yes |
| Have you changed your ongoing medical treatment during the past month? | □ No, I have not changed my medical treatment; □ Yes, I have had problems obtaining my usual treatment; □ Yes, I have stopped my treatment (for example corticosteroids, anti-inflammatory drugs) so as not to risk aggravating a potential Coronavirus infection; □ Yes, for another reason | |
| Have you been afraid of getting infected with the Coronavirus (SARS-CoV-2) by going for treatment? | □ Yes, very afraid; □ Yes, a bit afraid; □ No, not very afraid;  □ No,barely afraid; □ No, not afraid | |
| Why did you have problems obtaining your usual treatment? | □ My appointment was postponed or cancelled; □ The health professional had closed his office; □ I could not have my children looked after to go for treatment; □ Other reason | |
| Please specify other reason. | ______________________________________ | |
| Why have you stopped your treatment? | □ Because I was too afraid of being infected during treatment.; □ Because I wanted to reduce my trips outside the home.; □ Because I wanted to protect other members in my family from getting infected.; □ Other reason | |
| Please specify other reason. | ______________________________________ | |
| During the past month, did you need to see a health professional for an acute health problem not related to the Coronavirus (SARS-CoV-2) and not part of an ongoing treatment? | □ No; □ Yes | |
| If yes, how did this consultation take place? | □ By phone or telemedicine; □ At home ; □ In a doctor's office; □ In a  hospital department; □ In hospital emergency rooms; □ Other | |
| Have you been afraid of being infected with the  Coronavirus (SARS-CoV-2) during the consultation? | □ Yes, very afraid; □ Yes, a bit afraid; □ No, not very afraid;  □ No, barely afraid; □ No, not afraid | |
| Please specify other reason. | ______________________________________ | |
| The SwissCovid App has been launched by the Swiss Federal Office of Public Health to warn smartphone users in case of possible exposure risks. The app records, if a contact has been in close proximity of 1.5m or less for longer than 15 minutes. If an app user tested positive for the Coronavirus, she or he can anonymously notify other app users, who were in close proximity during the infectious period. |  | |
| Are you using the SwissCovid App? | □Yes, permanently; □Yes, but sometimes I turn off Bluetooth to pause the SwissCovid App; 3 No, but I am planning to use it; □No; □No, I have uninstalled the App | |
| Why are you currently not using the SwissCovid App? | □ I have not heard about the app; □I don't think the app is useful for me; □I can't install the app (e.g., owing to technical difficulties or because I do not own an Android or iOS smartphone); □I fear for my privacy and protection of my data; □Other reasons | |
| Please specify other reason. |  | |
| Were you ever notified by the SwissCovid App that you have been in close proximity to a Corona-positive person? | □No, I have never received a notification; □Yes, I called the  recommended Infoline SwissCovid; □Yes, I undertook other steps; □Yes, but I did not undertake any steps | |
| Which steps? | ______________________________________ | |
| What motivates you to be part of Corona Immunitas?  What is your experience with our study? | ______________________________________ | |
| How does the Covid-19 pandemic currently affect your daily life? Does the Corona Immunitas study cover these aspects well enough? | ______________________________________ | |
| Over the past 30 days, have you been vaccinated (fully or partly) against the coronavirus? | □Yes, I have been vaccinated in the past 30 days; □Yes, but I have been vaccinated more than 30 days ago; □No, I have not been vaccinated at all so far; □I don't want to answer; □Yes, and I have already reported all vaccination doses I have received so far | |
| Up to now, have you been advised regarding the Coronavirus vaccination? | □Yes, I was advised to get vaccinated; □Yes, I was advised not to get  vaccinated; □No, I was not advised | |
| Who advised you to get vaccinated? | □FOPH / Cantonal Medical Officer; □Personal physician; □Another  healthcare professional; □Employer; □Friend / Family member;  □ Religious leader; □Other | |
| Please specify | ______________________________________ | |
| Who advised you to not get vaccinated? | □FOPH / Cantonal Medical Officer; □Personal physician; □Another  healthcare professional; □Employer; □Friend / Family member;  □ Religious leader; □Other | |
| Please specify | ______________________________________ | |
| Why did you get vaccinated against the Coronavirus? (check all that apply) | □ I have a chronic disease or disorder; □I am at risk because of my age; □I work in the healthcare system; □I live with or look after one or more vulnerable person(s); □I am vulnerable with regard to the Coronavirus for some other reason than the above please specify; □I want to protect myself; □I want to get back to normal life as fast as possible; □My employer wants me to be vaccinated; □I want to travel (for work or leisure) and need or want an immunization certificate; □I want to contribute to the protection of my community and / or society; □Other reason(s) | |
| Please specify | ______________________________________ | |
| Which coronavirus vaccine did you receive? | □PfizerBioNTech (Comirnaty®, BNT162b2); □Moderna (mRNA-1273); □AstraZeneca / Oxford (AZD1222); □Janssen / Johnson & Johnson  (Ad26.COV2.S.); □Other; □I don't know; □Novavax (NVX-CoV2373); □CureVac (CVnCoV) | |
| Please specify | ______________________________________ | |
| How many doses of the coronavirus vaccine have you | | □ 1; □ 2; □ 3; □ Other |
| Please specify | | ______________________________________ |
| When did you receive the first vaccination dose? | | ______________________________________ |
| When did you receive the second vaccination? | | ______________________________________ |
| After the vaccination, did you experience any unwanted effects or symptoms that may have been related to the injection and / or the vaccine? | | □Yes; □Yes, but unsure if it is related to the vaccine; □No; □I have  already reported all unwanted effects and symptoms |
| Describe the unwanted effects or symptoms | | ______________________________________ |
| Date when unwanted effects or symptoms started (in case of several symptoms, please reference the most serious ones) | | ______________________________________ |
| How severe were the unwanted effects or symptoms (in case of several symptoms, please reference the most serious ones)? | | □Self-limiting; □I needed medical treatment.; □Physician or other  healthcare professional office / clinic / emergency room visit;  □ Hospitalization; □ Other |
| Number of days (if known) | | ______________________________________ |
| Please specify | | ______________________________________ |
| What is the state of the unwanted effects or symptoms at the time of reporting (in case of several symptoms, please reference the most serious ones)? | | □Recovered; □Improving; □Not recovered; □Unknown |
| Treatment of the unwanted effects or symptoms (if any) | | ______________________________________ |
| Was / were the unwanted effects or symptom(s) you experienced officially recognized by your physician or other health professional as a potential vaccine side effect? | | □Yes; □No; □I don't know |
| Do you think you are currently eligible to receive the Coronavirus vaccine? | | □No, I am not eligible; □I don’t know if I am eligible or not;  □Yes, I am eligible |
| Do you have an appointment for the coronavirus vaccination? | | □Yes, I have an appointment and will be vaccinated soon; □Yes, I have an appointment, but I have difficulties reaching the vaccination facility; □No, I don't have an appointment because I have difficulties scheduling an appointment; □No, I don't have an appointment for other reasons; □ No, I don't have an appointment because I am not eligible yet |
| Please specify | | ______________________________________ |
| Thinking about vaccination in general, would you say you are personally | | 1 Strongly against, ..., 5 Strongly support |
| In answering the following questions, please refer to vaccination in general. Please indicate the degree to  which you agree or disagree with the following statements: | |  |
| I generally trust vaccine manufacturers or pharmaceutical companies | | 1 Strongly disagree, ..., 5 Strongly agree |
| I generally trust the Federal Office of Public Health (FOPH) / Bundesamt für Gesundheit (BAG) | | 1 Strongly disagree, ..., 5 Strongly agree |
| I understand how vaccination helps my body fight infectious diseases | | 1 Strongly disagree, ..., 5 Strongly agree |
| I feel it is important that I get vaccinated | | 1 Strongly disagree, ..., 5 Strongly agree |
| Vaccination forms part of a healthy lifestyle | | 1 Strongly disagree, ..., 5 Strongly agree |
| Once the coronavirus vaccine is available to you, how likely is it that you will decide to get vaccinated? | | 1 not at all likely, …, 5 very likely |
| Why do you want to or need to get vaccinated against the Coronavirus? (check all that apply) | | □I have a chronic disease or disorder; □I am at risk because of my age; □I work in the healthcare system; □I live with or look after one or more vulnerable person(s); □I am vulnerable to the Coronavirus for some other reason than the above; □I want to protect myself ; □I want to get back to normal life as fast as possible; □My employer wants me to be vaccinated; □I want / need to travel (for work or leisure) and want / need or want an immunization certificate; □I want to contribute to the protection of my community and / or society; □Other |
| Please specify | | ______________________________________ |
| In answering the following questions, please refer to the coronavirus vaccine / vaccination or the Coronavirus disease. Please indicate the degree to  which you agree or disagree with the following statements related to the coronavirus vaccine | |  |
| I prefer to wait before being vaccinated until more is known about how effective the vaccine is | | 1 Strongly disagree, ..., 5 Strongly agree |
| I prefer to wait before being vaccinated until more is known about the vaccine's safety | | 1 Strongly disagree, ..., 5 Strongly agree |
| I believe that vaccination protects me from an infection with the coronavirus | | 1 Strongly disagree, ..., 5 Strongly agree |
| I believe that the vaccination protects me against a severe course of coronavirus infection | | 1 Strongly disagree, ..., 5 Strongly agree |
| I believe that the vaccination protects against  transmission of the coronavirus to others | | 1 Strongly disagree, ..., 5 Strongly agree |
| I am afraid of possible side effects | | 1 Strongly disagree, ..., 5 Strongly agree |
| I follow what my religious faith prescribes regarding this vaccination | | 1 Strongly disagree, ..., 5 Strongly agree |
| I prefer natural immunity against the coronavirus to vaccine-induced immunity | | 1 Strongly disagree, ..., 5 Strongly agree |
| I prefer natural or traditional remedies to the disease rather than being vaccinated | | 1 Strongly disagree, ..., 5 Strongly agree |
| I am afraid of injections | | 1 Strongly disagree, ..., 5 Strongly agree |
| I am concerned about getting infected if I go to a clinic where vaccinations are administered | | 1 Strongly disagree, ..., 5 Strongly agree |
| I would rather protect myself by other means (physical distancing, hand hygiene, wearing a mask) than be vaccinated | | 1 Strongly disagree, ..., 5 Strongly agree |
| I think that the vaccine will provide long-lasting immunity | | 1 Strongly disagree, ..., 5 Strongly agree |
| I want to protect myself | | 1 Strongly disagree, ..., 5 Strongly agree |
| I want to contribute to the protection of my community / society | | 1 Strongly disagree, ..., 5 Strongly agree |
| I want to contribute to the protection of someone I know who is vulnerable | | 1 Strongly disagree, ..., 5 Strongly agree |
| I want to get back to a normal life as fast as possible | | 1 Strongly disagree, ..., 5 Strongly agree |
| I prefer to let those who will benefit most have first access to the vaccine | | 1 Strongly disagree, ..., 5 Strongly agree |
| Medical reasons (e.g., allergies) prevent me from being vaccinated | | 1 Strongly disagree, ..., 5 Strongly agree |
| I base my vaccination decision on the results of my serological test | | 1 Strongly disagree, ..., 5 Strongly agree |
| The coronavirus vaccine has been developed too quickly | | 1 Strongly disagree, ..., 5 Strongly agree |
| I feel overwhelmed by information on the coronavirus vaccine | | 1 Strongly disagree, ..., 5 Strongly agree |

Note: Questions used in the analysis of this manuscript are marked with a grey background
